# Assessment of Hepatocellular Carcinoma Awareness and Understanding Among Health Science Students: A Cross-Sectional Study

**DOI:** 10.3390/healthcare13212669

**Published:** 2025-10-23

**Authors:** Zaki H. Hakami

**Affiliations:** Department of Medical Laboratory Technology, College of Nursing and Health Sciences, Jazan University, Jazan 45142, Saudi Arabia; zakih@jazanu.edu.sa; Tel.: +966-553102418

**Keywords:** hepatocellular carcinoma, awareness, health science students, cross-sectional study

## Abstract

**Background:** Hepatocellular carcinoma (HCC) is a prominent contributor to global cancer-related mortality and is characterized by unfavorable prognosis despite regional discrepancies in its occurrence. Understanding and awareness of HCC among health science students are crucial for early detection and enhanced patient outcomes. **Methods**: This cross-sectional study evaluated awareness of HCC among health science students at Jazan University and identified areas that require further education. The study included health science students enrolled in various academic programs at Jazan University. A structured online questionnaire was used to collect demographic information and assess knowledge related to HCC. The sample size was determined based on prevalence estimates, and statistical analyses were performed using the R software (version 4.3.1). **Results**: The study found that 61% of the health science students had good knowledge of HCC. Of the 411 participants, most were young (≤24 years), single, and enrolled in allied and health sciences programs. Although 55.20% were familiar with HCC, their awareness of screening methods and preventive measures was limited. Hepatitis B vaccination has been recognized as an effective preventive measure. A logistic regression analysis revealed significant associations between age, sex, academic year, and awareness of HCC, with 1.91-, 1.94-, and 2.83-times higher odds ratios, respectively. **Conclusions**: This study underscores the need for targeted educational interventions and public awareness campaigns to improve understanding, early detection, and prevention of HCC among health science students.

## 1. Introduction

Liver cancer, particularly HCC, is a significant global public health issue. It is the sixth most prevalent cancer worldwide and the fourth leading cause of cancer-related death [1]. HCC is a primary liver cancer that originates from hepatocytes, the primary functional cell type in the liver [2]. This malignancy is characterized by uncontrolled proliferation of hepatocytes, resulting in the development of malignant tumors within the liver. It is the most prevalent histological subtype of liver cancer, accounting for approximately 90% of all liver cancer cases [3].

**Figure 1:**
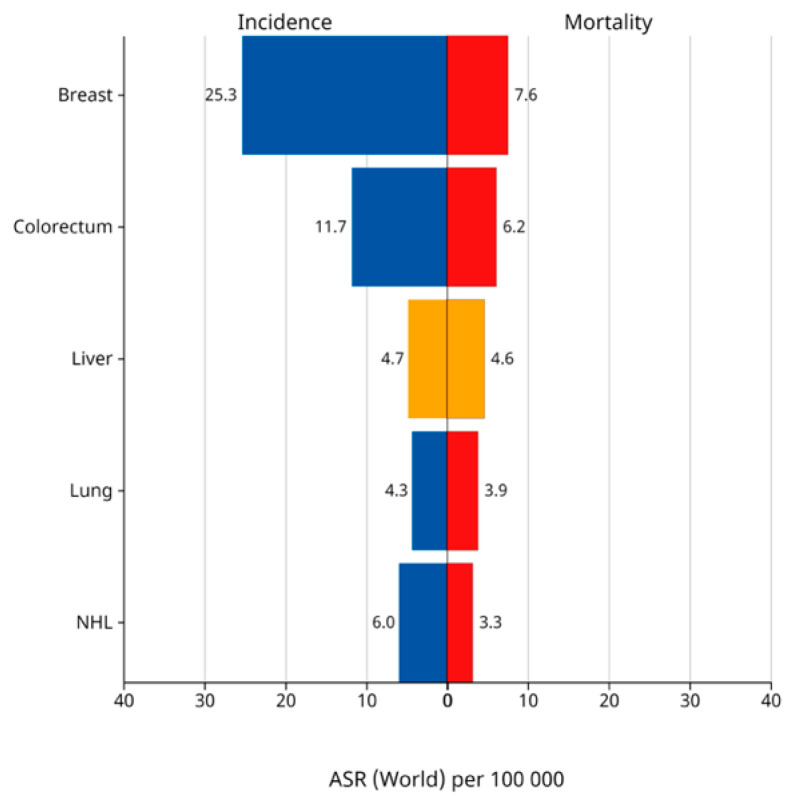
Incidence and mortality of liver cancer in Saudi Arabia. This figure provides concise information about age-standardized rates for both incidence and mortality, categorized by sex (male and female), in Saudi Arabia for the year 2022. These rates are expressed per 100,000 individuals, allowing for comparisons across populations with different age distributions. International Agency for Research on Cancer, Cancer Today, 2024. Age-Standardized Rate per 100,000, Incidence and Mortality, Both sexes, in 2022, Saudi Arabia. Globocan 2022 (version 1.1). Available on: https://gco.iarc.fr/today/en/dataviz/bars?types=0_1&mode=cancer&group_populations=1&populations=682&sort_by=value1, accessed on 20 January 2025 [25].

The epidemiology of HCC is marked by dynamic shifts in incidence rates across different regions [4]. While historically high-incidence areas have seen declining rates, low-incidence regions, such as India, the Americas, Oceania, and parts of Europe, have experienced rising rates, reflecting changes in risk factors and healthcare practices [5]. Despite regional variations, the prognosis of HCC remains grim globally, with similar incidence and mortality rates [4]. In 2018, the global incidence and mortality rates were 9.3 and 8.5 per 100,000 person-years, respectively, underscoring the severe impact of this disease on individuals and healthcare systems worldwide [6]. When considering sex disparities, men were significantly more susceptible to HCC than women, with a global incidence ratio of 2.8:1 [7]. In Saudi Arabia, liver cancer ranks seventh among males and tenth among females in cancer rankings in 2020, with 450 cases, accounting for 4.7% of all diagnosed cancer cases among Saudi nationals (Figure 1). Among those affected, 291 (64.7%) were males, while 159 (35.3%) were females, resulting in a male-to-female ratio of 183 to 100 [8]. These statistics highlight the varying sex prevalence and overall burden of liver cancer among the Saudi Arabian population.

HCC is a complex malignancy driven by various risk factors, including chronic viral infections (hepatitis B and C), heavy alcohol consumption, non-alcoholic fatty liver disease, and metabolic syndromes, such as obesity and diabetes [9,10,11]. Its pathogenesis involves molecular and cellular events, with chronic inflammation and liver fibrosis often serving as intermediaries [12]. Genetic and molecular alterations, including DNA mutations, epigenetic modifications, and disrupted cellular signaling pathways, drive malignant hepatocyte proliferation and survival [13]. Notable molecular alterations include mutations in the TERT promoter and tumor suppressor gene p53 [14,15]. Moreover, chronic liver inflammation and endoplasmic reticulum stress triggered by misfolded proteins or metabolic dysregulation create a conducive microenvironment for HCC development [16,17,18]. Abnormalities in cell signaling pathways, such as the Wnt/β-catenin and PI3K-AKT-mTOR pathways, are also critical drivers of uncontrolled cell growth and HCC progression [19,20].

Insufficient awareness of HCC is a notable contributor to its rising incidence, underscoring the need for enhanced education and knowledge dissemination [21,22]. Assessing awareness and knowledge of HCC among health science students is crucial because of its growing significance in global healthcare. HCC poses unique challenges, such as late-stage diagnosis [23] and limited treatment options [24]. Medical and allied health students play a crucial role in this endeavor by serving as future frontline healthcare providers. Their knowledge and awareness of HCC can facilitate early recognition of risk factors, symptoms, and appropriate screening strategies, ultimately contributing to a reduction in HCC incidence and mortality rates. Moreover, their engagement in educational initiatives and community outreach programs can enhance public awareness and promote preventive measures against HCC.

Therefore, this study aimed to evaluate the current awareness and knowledge of HCC among health science students at Jazan University, identify areas for further education, and emphasize the pivotal role of medical education in combating this increasingly prevalent and life-threatening cancer.

## 2. Materials and Methods

### 2.1. Study Design

This study employed a cross-sectional design to assess awareness and knowledge of HCC among health science students at Jazan University.

### 2.2. Study Setting and Population

This study was conducted at Jazan University, focusing on the population of health science students enrolled in six distinct academic programs. These programs encompass various academic levels, and are within the Faculty of Medicine, Faculty of Dentistry, Faculty of Pharmacy, Faculty of Applied Medical Sciences, Faculty of Nursing, and the Faculty of Public Health and Tropical Medicine.

### 2.3. Inclusion and Exclusion Criteria

The study included students enrolled in medical and allied health sciences programs, while excluding non-health science students enrolled in other disciplines or health science students who were not affiliated with Jazan University.

### 2.4. Sample Size

In a study conducted in Saudi Arabia [26], the prevalence of good HCC knowledge was 61%. We determined the sample size using a two-sided test with a significance level (α) of 10%, power (1 − β) of 80%, and an allowable margin of error in the proportion of HCC awareness (|P_1_ − P_0_|) of 6%. The sample size was calculated using the following equation:

[n = ((Z_(1−∝/2)_ + Z_(1−β)_)/(⌈P_1_ − P_0_ ⌉⁄√(P_(0)_ (1−P_0_))))]^2^ (1)

where α is the selected level of significance (10%), Z_1−α/2_ is 1.645 at the 10% level of significance, 1 − β is the selected power (80%), Z_1−β_ is 0.8416 at 80% power from the standard normal distribution, and |P_1_ − P_0_| is the allowable margin of error at 6%. The sample size was 411 participants.

### 2.5. Data Collection Tool

Data were collected through online questionnaires comprising 8 sections and 23 questions. The sections encompassed informed consent, demographic information, general knowledge of HCC, symptoms and risk factors, diagnostic tools, preventive measures, treatment, and knowledge sources. To achieve the objective of this study, participants’ awareness of HCC was divided into two categories (good and poor) based on their self-reported perception of the item and how they rated their awareness of HCC. The validation process for the questionnaire involved multiple steps to ensure its reliability and validity. The initial questionnaire was developed based on a thorough review of the existing literature on HCC awareness, symptoms, risk factors, and management [27,28]. Subsequently, a pilot study was conducted with a cohort of health science students (n = 20), distinct from the main study participants, to scrutinize the questionnaire’s clarity and comprehensibility. To measure the reliability of the questionnaire, the internal consistency was evaluated using Cronbach’s alpha. The results indicated good internal consistency, with a Cronbach’s alpha value of >0.82.

### 2.6. Statistical Analysis

Sampling weights were applied to the data to equalize the sampling distribution. Confounding factors included age, sex, marital status, residency, and education level. All confounding factors were weighted to ensure that our sample was representative of the standard normal population [29,30]. Qualitative variables are presented as frequencies and percentages. Associations between sociodemographic variables and HCC awareness were assessed using a logistic regression analysis. Odds ratios (ORs) and 95% confidence intervals (CIs) were calculated. All reported *p* values are two-sided, and statistical significance was set at α = 0.05. Analyses were conducted using the R software (version 4.3.1) for Windows and ggplot2.

### 2.7. Ethical Approval and Consent

This study adhered to the ethical principles outlined in the Declaration of Helsinki and was conducted in accordance with the protocols established by the Ethical Committee of Jazan University. This study was ethically approved with the reference number REC-45/03/792. Informed consent was obtained from all participants using an online consent form.

## 3. Results

### Number of Participants Invited to the Final Sample

**Figure 2:**
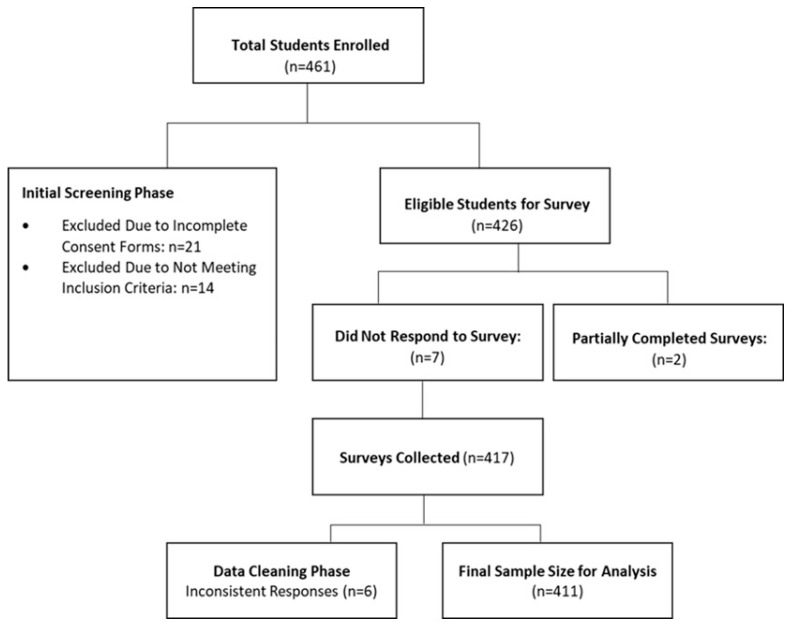
This flow diagram effectively illustrates the process and reasons for non-participation and exclusion in the study.

The study initially enrolled 461 students. After the initial screening phase, 35 students were excluded, with 21 being excluded due to incomplete consent forms and 14 for not meeting the inclusion criteria. Subsequently, 426 students were deemed eligible for the survey. Of these eligible participants, 7 did not respond to the survey, and 2 provided partially completed surveys, resulting in 417 surveys collected. During the subsequent data cleaning phase, six surveys were excluded due to inconsistent responses. Consequently, the final sample size for analysis comprised 411 students, as depicted in the flowchart below. The meticulous screening and cleaning processes underscore the rigor of our methodology, aiming to produce robust and reliable results from the data collected. These steps are imperative to uphold the integrity of the study and ensure that the findings are grounded in accurate and consistent participant information (Figure 2).

A total of 411 participants were included in the study; their demographic characteristics are presented in Table 1. The majority of participants were aged 24 years or younger, with 91.48% falling into this category. The sex distribution in this study was relatively balanced, with 55.20% identifying as female and 44.80% as male. The participants were predominantly single (85.10%) and lived in rural areas (58.30%). Nearly all participants were undergraduates (99.00%), and most were enrolled in allied and health sciences programs (87.70%). The distribution across the years of study revealed a varied representation, with the highest percentage in the 2nd year (37.30%) and the 4th year or beyond (33.50%). Notably, a large proportion of participants (93.50%) reported no family or friends with liver cirrhosis or liver disease. Conversely, 24.00% of participants reported a history of HCC or other cancers among their family or friends.

The data presented in Table 2 illustrate the responses of the surveyed participants regarding their knowledge and perceptions of HCC. Among the 411 respondents, the majority (92.60%) reported an awareness of the primary functions of the liver, indicating a basic understanding of liver physiology. Regarding familiarity with HCC, 55.20% of the participants had heard of it before the survey. However, only 35.80% of patients were aware of the screening methods for HCC. Among the screening tools mentioned, liver function tests were most common (66.40%). Additionally, while a significant proportion of participants believed in the preventability of HCC (57.80%), only 48.20% were familiar with preventive measures, with hepatitis B vaccination being the most recognized (76.50%). The study also revealed varying perceptions regarding the appropriate timing of HCC screening, with a high proportion (40.20%) believing that screening should occur when signs and symptoms manifest. Furthermore, the overwhelming majority (96.70%) supported public awareness campaigns for early detection and prevention. Despite this awareness, uncertainty persists regarding treatment options, with 41.70% expressing doubts about HCC’s treatability. Nonetheless, a notable proportion (44.30%) indicated an awareness of all treatment modalities mentioned, with chemotherapy, surgery, and liver transplantation being the most recognized. Notably, curriculum/textbooks emerged as the primary source of knowledge acquisition (49.50%), followed by websites (22.10%) and TV programs and social media (14.90%). These findings underscore the importance of educational initiatives and targeted awareness campaigns to improve the understanding and mitigation of the HCC burden.

The results of the binary logistic regression analysis, as presented in Table 3, demonstrated the associations between the demographic variables and awareness of HCC. Gender played a crucial role, with males exhibiting significantly higher odds of good awareness (OR: 1.94, 95% CI: 1.24–3.03, *p* < 0.01) than females. However, marital status, residency, education level, and personal history of liver disease or cancer were not significantly associated with HCC awareness. The year of study, however, showed significant associations, with third-year students exhibiting 1.94 times higher odds (95% CI: 0.89–4.21) and students in the fourth year or above demonstrating 2.83 times higher odds (95% CI: 1.39–5.77) of having good awareness than first-year students. These results confirm the potential association between a higher academic year and increased awareness of HCC.

**Figure 3:**
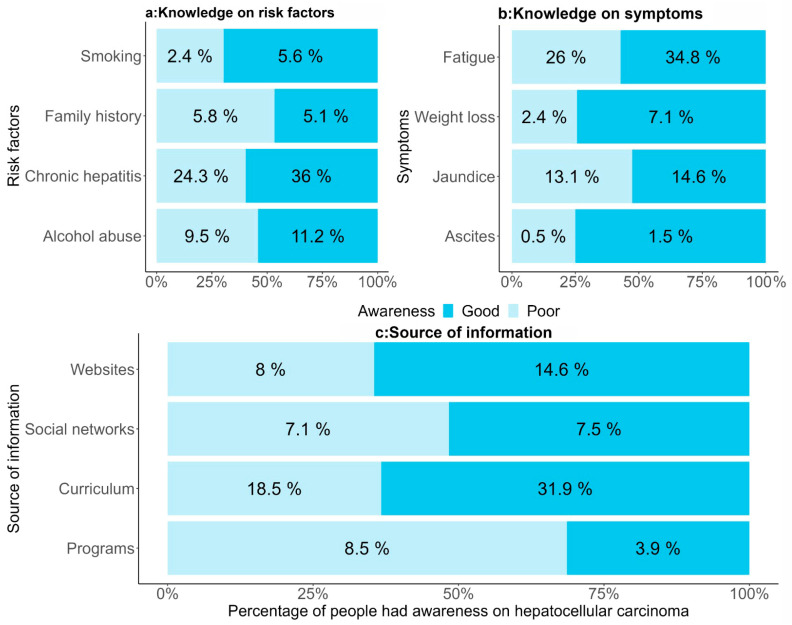
Awareness of hepatocellular carcinoma among students. (**a**) More than half of the students (60.3%) reported that the most common risk factor for HCC was chronic hepatitis, followed by alcohol abuse (20.7%). (**b**) More than half of the students (60.8%) stated that the most common symptom of HCC is fatigue, followed by jaundice (9.5%). (**c**) More than half of the students (50.4%) stated that their knowledge of HCC was obtained from the curriculum, of which a major portion (31.9%) had good awareness; followed by knowledge obtained from websites (22.6%), of which 14.6% had good awareness.

The results illustrated in Figure 3 underscore the prevalent understanding among the surveyed students regarding the significant risk factors and symptoms associated with HCC. Notably, chronic hepatitis emerged as the primary risk factor, as acknowledged by 60.3% of respondents. Additionally, alcohol abuse was recognized by 20.7% of students as another prevalent risk factor contributing to the development of HCC. Regarding symptoms, fatigue emerged as the most commonly recognized indicator, with 60.8% of students associating it with HCC. Jaundice was the second most recognized symptom, cited by 9.5% of the respondents. Furthermore, this study examined the various channels through which students acquire knowledge about HCC. This revealed that a majority of students (50.4%) derive their understanding from academic curricula, with 31.9% demonstrating commendable awareness levels. Websites also play a significant role in knowledge acquisition, with 22.6% of the students utilizing online platforms for information, among which 14.6% exhibited a commendable level of awareness.

## 4. Discussion

This study aimed to investigate the sociodemographic characteristics and perceptions of HCC among 411 participants. These findings provide valuable insights into the study population and shed light on areas that require attention to improve awareness and understanding of HCC. The gender distribution was balanced, with 55.20% of participants identifying as female and 44.80% as male. Additionally, the majority of participants were undergraduate students enrolled in allied health and health sciences programs. This result aligns with previous studies that have highlighted the importance of targeting educational interventions towards younger populations, particularly those in healthcare-related fields, to improve awareness and understanding of HCC [21,31]. In terms of HCC awareness, more than half of the participants (55.20%) had heard of HCC before the survey, indicating a moderate level of familiarity with the disease. However, only a minority of the participants were aware of HCC screening methods, suggesting a knowledge gap in this area. The study findings also revealed varying levels of awareness regarding preventive measures for HCC. While a significant proportion of participants (57.80%) believed in the preventability of HCC, less than half were familiar with specific preventive measures. In a related context, some studies have underscored the importance of hepatitis B vaccination in HCC prevention [7,32]. Our findings corroborate this finding, as hepatitis B vaccination was the most recognized preventive measure, indicating some awareness of the association between hepatitis B infection and HCC.

Previous studies have indicated that age, sex, and education are significant factors influencing the awareness levels of various health conditions, including cancer [33,34]. Our study, employing binary logistic regression analysis, confirmed these associations, revealing that individuals aged >24 years and males have significantly higher odds of having a good awareness of HCC than their counterparts. Additionally, there was a significant association between academic year and awareness of HCC, with higher academic years being linked to increased awareness. The medical curriculum is divided into basic sciences during the first three years, followed by clinical training in the 4th and 5th years. Given this structure, 4th- and 5th-year students were grouped together, as they transition into the clinical phase with more hands-on patient interaction. This distinction may influence their awareness of HCC differently compared to students in the earlier basic science years. These findings are consistent with those of other studies that have shown that formal education and exposure to healthcare-related subjects are associated with improved awareness [35,36]. Thus, integrating HCC-related content into healthcare curricula and providing targeted educational interventions can enhance knowledge and awareness among students, which can have long-term benefits in combating many diseases, including HCC.

In addition, a significant proportion of participants (41.70%) expressed doubts about the treatability of HCC. This finding indicates the need for further education and information dissemination regarding treatment options for HCC. Healthcare professionals, patient advocacy groups, and educational institutions can play a crucial role in providing accurate and up-to-date information regarding advancements in HCC treatment modalities, including chemotherapy, surgery, liver transplantation, and targeted therapies. Increasing knowledge of treatment options can empower individuals to make informed decisions and engage in shared decision making with healthcare providers [35].

Numerous scientific studies have consistently demonstrated the effectiveness of public awareness initiatives in enhancing cancer-related knowledge, encouraging early detection practices, and promoting healthy behavioral patterns [21,37]. The results of the current study indicate that a large percentage of the participants (96.70%) supported public awareness campaigns aimed at early detection and prevention of HCC. These findings underscore the need for public awareness campaigns to enhance understanding and encourage proactive health care practices. Utilizing diverse channels such as traditional media, social media, and community outreach programs, public awareness campaigns can effectively distribute information on HCC risk factors, symptoms, screening methods, and preventive measures. To maximize their effectiveness, these campaigns should be culturally sensitive, accessible, and tailored to the specific requirements of the target population.

Unlike a previous study in southern Saudi Arabia [26] that examined public awareness, our research uniquely focuses on health science students—future frontline healthcare providers—by assessing their knowledge of HCC, identifying educational gaps, and analyzing key sociodemographic predictors of awareness. This study contributes to the literature by proposing targeted, curriculum-based interventions aimed at enhancing early detection and prevention efforts, thereby strengthening the role of future healthcare professionals in combating HCC.

### 4.1. Study Limitations

One notable limitation of this study is the sampling method and generalizability of the findings. The study population consisted mainly of undergraduate students, particularly those enrolled in allied and health sciences programs. This highly specific sample may not be representative of the broader population and the findings may not be applicable to other age groups, educational backgrounds, or geographic locations. Therefore, caution should be exercised when generalizing the results beyond the study population. Additionally, reliance on self-reported data introduces the potential for recall and social desirability biases, which may influence the accuracy of the responses.

Despite these limitations, the findings of this study have important implications for public health interventions aimed at increasing the awareness, early detection, and prevention of HCC. This study highlighted the need for targeted awareness campaigns to address specific knowledge gaps and misconceptions identified in the study population. Public health initiatives should prioritize raising awareness about HCC, promoting preventive measures, such as hepatitis B vaccination, and educating the public about early detection through regular screening. Collaboration among health care providers, educational institutions, policymakers, and community organizations is vital for effective interventions to reduce the burden of HCC.

### 4.2. Future Directions

This study provides the foundation for further research in several areas. Future studies should explore the reasons behind age and sex disparities in HCC awareness to develop more targeted interventions. Research should also investigate the impact of cultural and socioeconomic factors on knowledge and awareness of HCC. Longitudinal studies can assess the effectiveness of educational interventions and public health campaigns in improving awareness, early detection rates, and health outcomes related to HCC. Additionally, this research could focus on other at-risk populations, such as individuals with a family history of HCC or specific ethnic groups with a higher HCC prevalence, to gain a comprehensive understanding of awareness levels and develop tailored interventions.

## 5. Conclusions

This study offers valuable insights into the sociodemographic factors, knowledge gaps, and awareness levels related to HCC within the study population. The most prominent finding is the critical role of accessible, accurate information, as persistent misconceptions and gaps in knowledge highlight the urgent need for enhanced education and awareness regarding HCC risk factors, prevention, screening, and treatment. These disparities emphasize the importance of tailored educational initiatives and targeted public health campaigns to promote early detection and effective prevention. Furthermore, integrating HCC-related content into medical education, through innovative teaching strategies, case-based learning, and interprofessional collaboration, can strengthen health science students’ competency and ultimately improve patient outcomes in HCC management and care.

## Figures and Tables

**Table 1:** Background characteristics of the participants.

Variable		All Participants (n = 411)		
		Weighted	%	Unweighted
Age	≤24	376	91.48%	377
	>24	35	8.52%	34
Gender	Female	227	55.20%	230
	Male	184	44.80%	181
Marital status	Single	350	85.10%	349
	Married	61	14.90%	62
Residency	Rural	239	58.30%	236
	Urban	172	41.70%	175
Education level	Undergraduate	407	99.00%	407
	Post graduate	4	1.00%	4
College/program of study	Medical/Dental	51	12.30%	52
	Allied and Health Sciences	360	87.70%	359
Year of study	1st	48	11.60%	47
	2nd	153	37.30%	145
	3rd	72	17.50%	74
	≥4th	138	33.50%	145
Have any of your family or friends had liver cirrhosis or any liver diseases?	No	384	93.50%	384
	Yes	27	6.50%	27
Have any of your family or friends had hepatocellular cancer or any other cancer?	No	312	76.00%	313
	Yes	99	24.00%	98

**Table 2:** Knowledge and awareness on hepatocellular cancers and other cancers.

Variable		All Participants (n = 411)		
		Weighted	%	Unweighted
Do you know the primary functions of the liver?	No	30	7.40%	29
	Yes	381	92.60%	382
Have you heard of hepatocellular cancer before taking this survey?	No	184	44.80%	177
	Yes	227	55.20%	234
Are you aware of the screening methods used to detect hepatocellular cancer?	No	264	64.20%	251
	Yes	147	35.80%	160
Which of the following screening tools do you believe could be employed for the detection of hepatocellular carcinoma? (Select all that apply)	Alpha-fetoprotein	5	1.10%	5
	CT and MRI Scan	68	16.40%	67
	Liver Biopsy	29	7.00%	28
	Liver function	273	66.40%	275
	Liver ultrasound	24	5.80%	24
	X-ray of the ab	13	3.20%	12
When do you believe it is appropriate for someone to undergo screening for hepatocellular carcinoma?	40–49 Years	74	17.90%	76
	50–59 Years	50	12.10%	51
	≥60 Years	29	6.90%	30
	When risk factors present	94	22.80%	94
	When signs & symptoms appear	165	40.20%	160
Do you believe that Hepatocellular Carcinoma can be prevented?	No	12	2.90%	12
	I don’t know	161	39.30%	158
	Yes	238	57.80%	241
Are you familiar with any preventive measures to reduce the risk of hepatocellular cancer?	No	213	51.80%	203
	Yes	198	48.20%	208
Which of the following are effective preventive measures for hepatocellular cancer? (Select all that apply)	Hepatitis B Vaccination	314	76.50%	312
	No Alcohol/Smoking	79	19.20%	82
	Regular Exercise	16	3.80%	15
	Regular liver check-up	2	0.50%	2
Do you believe public awareness campaigns about Hepatocellular cancer are essential for early detection and prevention?	No	14	3.30%	14
	Yes	397	96.70%	397
Do you think hepatocellular cancer is treatable?	No	31	7.60%	31
	I don’t know	171	41.70%	165
	Yes	208	50.70%	215
Which of the following treatment options for hepatocellular carcinoma you are aware of?	Chemotherapy	86	21.00%	86
	Radiationtherapy	21	5.20%	21
	Immunizationtherapy	4	1.00%	4
	Liver transplantation	51	12.40%	52
	Surgery	66	16.10%	69
	All of the above	182	44.30%	179
Where did you acquire most of your knowledge about hepatocellular carcinoma?	Cancer Awareness	55	13.50%	51
	Curriculum/Text books	203	49.50%	207
	TV Programs/Social	61	14.90%	60
	Websites	91	22.10%	93

**Table 3:** Association between awareness of hepatocellular carcinoma and demographic characteristics.

Variable		All Participants (n = 411)	Awareness Good (n = 206)	OR ^§^	95% CI	
					LL	UL
Age	≤24	376	190	-	-	-
	>24	35	16	1.91	0.8	4.57
Gender	Female	227	124	-	-	-
	Male	184	82	1.94 **	1.24	3.03
Marital status	Single	350	173	-	-	-
	Married	61	33	1.07	0.55	2.06
Residency	Rural	239	109	-	-	-
	Urban	172	97	1.34	0.86	2.08
Education level	Undergraduate	407	204	-	-	-
	Post graduate	4	2	1.56	0.18	13.85
College/program of study	Medical/Dental	51	29	1.34	0.66	2.72
	Allied and Health Sciences	360	176	-	-	-
Year of study	1st	48	22	-	-	-
	2nd	153	50	0.61	0.31	1.21
	3rd	72	42	1.94 **	0.89	4.21
	≥4th	138	92	2.83 **	1.39	5.77
Have any of your family or friends had liver cirrhosis or any liver diseases?	No	384	192	-	-	-
	Yes	27	14	1.03	0.63	1.68
Have any of your family or friends had hepatocellular cancer or any other cancer?	No	312	158	-	-	-
	Yes	99	47	1.06	0.43	2.57

OR ^§^: odds ratio, CI: confidence interval, LL: lower limit, UL: upper limit. ** highly significant.

## Data Availability

The datasets generated and/or analyzed during the current study are available from the corresponding author upon reasonable request.
